# Quantification of Ions in Human Urine—A Review for Clinical Laboratories

**DOI:** 10.3390/biomedicines12081848

**Published:** 2024-08-14

**Authors:** Ana Rita Ferrão, Paula Pestana, Lígia Borges, Rita Palmeira-de-Oliveira, Ana Palmeira-de-Oliveira, José Martinez-de-Oliveira

**Affiliations:** 1 Centro Hospitalar Universitário Cova da Beira, EPE, 6200 Covilhã, Portugal; pgpestana@gmail.com; 2 Health Sciences Research Centre, Universidade da Beira Interior, 6201 Covilhã, Portugal; apo@fcsaude.ubi.pt (A.P.-d.-O.); jmo@fcsaude.ubi.pt (J.M.-d.-O.); 3 Labfit-HPRD, 6200 Covilhã, Portugal; ligia.borges@labfit.pt (L.B.); rpo@fcsaude.ubi.pt (R.P.-d.-O.)

**Keywords:** urine, ions, clinical laboratory tests

## Abstract

Urine is an organic fluid produced by the kidney, and its analysis is one of the most requested laboratory tests by clinicians. The ionic composition of urine has been shown to be a good health indicator: it is useful for the diagnosis of several diseases, as well as monitoring therapeutics. This review considers laboratorial techniques that have been used throughout time for the quantification of ions in urine, and also considers some methodologies that can potentially be used in clinical laboratories for this kind of analysis. Those methods include gravimetry, titration, flame emission spectrophotometry (flame photometry), fluorimetry, potentiometry (ion selective electrodes), ion chromatography, electrophoresis, kinetic colorimetric tests, enzymatic colorimetric tests, flow cytometry, atomic absorption, plasma atomic emission spectrometry, and paper-based devices. Sodium, potassium, chloride, calcium, and magnesium are among the most important physiological ions, and their determination is frequently requested in hospitals. There have been many advances regarding the analysis of these ions in 24 h urine. However, there is still some way to go concerning the importance of intracellular ions in this type of sample as well as the use of occasional urine for monitoring these parameters.

## 1. Urine in Diagnosis—An Overview

Urine is an organic fluid produced by the kidney. It accumulates in the bladder and is excreted through the urethra. Urine has the function of eliminating substances found in excess and that are toxic for the body; this excretion occurs by three basic processes in the nephron: filtration, reabsorption, and secretion [1]. Filtration takes place inside the renal corpuscle, where the blood pressure inside the capillaries of the glomerulus leaks into the renal capsule as a filtrate, with a similar composition as blood plasma, although with a smaller amount of protein, which goes towards the kidney’s tubules [1]. During reabsorption, some substances from the filtrate are reabsorbed into the blood, with about 65% of total sodium and water reabsorbed in the proximal tubule. Glucose and amino acids are almost completely reabsorbed [2]. Salts are reabsorbed at the Henle loop, and a high reabsorption of ions occurs at the distal tube [2]. During secretion, there is a transfer of molecules and ions from the blood to the nephron, like hydrogen, potassium, and ammonia [1].

Human urine is made up of about 3000 components, mostly water (about 95%), urea and uric acid, salts, and other substances [3]. Volume, acidity, and the concentration of salts in urine are regulated by hormones, such as antidiuretic hormone and aldosterone, which can also vary with the level of excretion, hydration, and protein and salts intake [3,4]. The most common electrolytes in urine are sodium, potassium, calcium, magnesium, chlorine, and phosphorus (in less quantity) [4]. Concerning the chemical compounds in urine, there is nitrogen (mostly in urea, the main product resulting from the metabolism of proteins), creatinine, and uric acid, as well as vitamins, hormones, and other organic compounds [3]. The presence of glucose, albumin, other proteins, bile pigments, or abnormal amounts of the usual constituents is usually an indicator of disease [3]. Urine is also a sterile fluid when expelled [3].

The presence of glucose in urine occurs mainly in diabetes mellitus [3]. The existence of blood in urine (haematuria and/or leukocyturia) may indicate urinary lithiasis, glomerulonephritis, cancer, or, more commonly, urinary tract infection [3]. Urinary infections develop when there are bacteria (bacteriuria) or fungus in the urine causing infection and can lead to the presence of pus in the urine (pyuria) [3].

## 2. Urine Analysis—A Summary

Urine analysis is one of the most requested laboratory tests by clinicians. It informs about the quantity, density, pH, physical appearance, presence of abnormal elements and bacteria, microscopic observation, and biochemical composition, such as urinary ions’ concentration [5].

The ionic composition of urine has been shown as a good indicator for the general state of patients’ health, and it is useful for the diagnosis of several diseases [5,6]. Electrolyte analysis in urine has an important value for kidney diseases, lithiasis, urinary tract infections, and cystic fibrosis, among others. In addition, daily assessment of electrolytes is useful for the acid-base monitoring of the body, as these are involved in most of the metabolic functions of the body [5].

## 3. Ions in Urine as Biomarkers

Sodium, potassium, and chlorine are among the most important physiological ions, and their concentration is requested more often in hospitals. They are mainly provided by food intake, absorbed in the gastrointestinal tract, and excreted through the kidneys [1].

Sodium is the main extracellular cation, responsible for maintaining fluid distribution and osmotic pressure. Among the causes of decreased sodium levels are vomiting or diarrhoea for long periods, decreased reabsorption in the kidneys, and excessive fluid retention. The most common causes for increased sodium include excessive fluid loss, high salt intake, and increased renal reabsorption [2].

Potassium is the main intracellular cation and is crucial for neurological and cellular activity [5]. Among the causes for decreased potassium levels, there is reduced potassium intake in the diet or excessive loss of potassium due to diarrhoea, prolonged vomiting, and increased renal excretion. Increasing levels of potassium can be caused by dehydration or shock, severe burns, ketoacidosis due to diabetes, and potassium retention by the kidneys [4].

Chloride is the main extracellular anion, and it functions as a regulator for the balance and distribution of extracellular fluids [2]. As with other ions, common causes of chloride decrease include reduced intake of food, prolonged vomiting, and reduced renal reabsorption, as well as the occurrence of some forms of acidosis and alkalosis [3]. There is an increase in chloride values in cases of dehydration, renal failure, some forms of acidosis, high intake of chloride, and poisoning with salicylate [3].

Laboratory determination for ionogram (sodium, potassium, and chloride) concentrations in patients must always be performed considering signs and symptoms, underlying pathologies, and therapeutics (diuretics, antihypertensives, etc.) as they promote changes in these parameters [6].

Reference values for adults refer to 24 h urine collections, in order to mitigate variations throughout the day: sodium—40 to 220 mmol/24 h; potassium—25 to 125 mmol/24 h; and chloride—110 to 250 mmol/24 h [5,7].

Calcium is the most abundant mineral element in the body, mostly present in bones (99%). The remaining calcium is distributed in various tissues and fluids. It is an extracellular ion, and it plays a crucial role for the maintenance of life: among the extra skeletal functions of calcium, it is also involved in blood coagulation, neuromuscular conduction, skeletal muscle and cardiac excitability, enzymatic activation, and in the preservation of the integrity and permeability of the cell membranes [3,5,8]. Calcium excretion is affected by protein entry and excretion of sodium. A low-sodium diet will decrease calcium excretion and vice versa. Changes in urinary calcium values are generally related to the presence of kidney stones or other kidney diseases, parathyroid pathologies, bone diseases, or other pathologies that lead to changes in blood calcium levels [5].

The calculated reference values for adults refer to 24 h urine collections: between 2.5 and 7.5 mmol/24 h [5,9].

Magnesium (as potassium) is one of the most important intracellular cations and a cofactor of many enzyme systems [3,10]. Thus, all adenosine triphosphate (ATP)-dependent enzymatic reactions require magnesium as a cofactor of the ATP–magnesium complex. Approximately 69% of magnesium is stored in the bones. The rest is part of the intermediate metabolism, with about 70% in free form and 30% bound to proteins, citrates, phosphates, and other complex forms [11]. Magnesium levels in serum are kept constant within very narrow limits, and its regulation is made mainly by the kidneys [11]. Changes in urine magnesium levels are usually related to the use of diuretics, variations in serum calcium, kidney dysfunction, alcoholism, changes in diet, pancreatitis, and diarrhoea. The calculated reference values for adults refer to 24 h urine collections, and it stands between 3.0 and 5.0 mmol/24 h [5,10,12].

This review aimed to investigate the variety of techniques used over time for the determination of ions in human samples, especially urine, with the purpose of developing auxiliary methodologies for urinary tract infections in further studies. We efforted to have a better understanding of the advantages and disadvantages of the different methodologies, hoping to identify which ones could be used nowadays to relate the quantification of some ions in urine (intra- or extracellular) and their eventual association with the existence and type of urinary tract infections in future investigations. With this, we hope to achieve an early diagnosis for urinary tract infections. We used PubMed database to search for key words such as “sodium, potassium, chloride, calcium and magnesium quantification” and “urine”. The preferred languages for this research were English, Portuguese, and Spanish.

## 4. Ions’ Quantification

Different methodologies can be used to obtain the concentration of different ions in urine. These techniques, which have evolved along time, can be described into 12 groups: gravimetry, titration, flame photometry, fluorimetry tests, potentiometry, chromatography, electrophoresis, kinetic colorimetric tests, enzymatic colorimetric tests, cytometry, atomic absorption, and, more recently, paper-based devices (rapid tests).

### 4.1. Gravimetry

This method was the first to be used at a laboratory to estimate the values of various ions in body fluids, in the 1940s. It is based on the precipitation of the component to be evaluated, drying the sample and weighing after various washing procedures [13]. The weight or mass obtained is converted to units per initial sample volume [13]. Later, it was also adapted to estimate intracellular potassium and sodium values in bacteria, comparing the weight and volume of the precipitate in dry suspensions [14].

Although it has the added value of being the first method to be used for this purpose, this procedure was very long and time-consuming, as it would take hours of work and preparation [13]. This method was used to measure potassium ions between 0.2 and 1.0 mg, although higher concentrations could be obtained with some precision by changing the washing methods [13].

### 4.2. Titration

The very first method described for measuring calcium and magnesium dates back to the 1940s, with the use of redox titrations for non-biological samples [15]. An indicator is added to the unknown solution (titrate). A known solution (titrant) reacts with the titrate as it is added [8]. The determination of the analyte can be performed directly or calculated with a titration curve [16].

The first titrant to be used for calcium measurement was permanganate [15]. In 1944, two Portuguese authors suggested the use of an indicator, capable of reacting with the release of iodine in the reaction, calling it “iodometric titration” [17]. This variation from the original titration enabled obtaining more accurate and sensitive results, with a smaller amount of sample to analyse [17]. Then, in 1946, ethylene-diamino-tetraacetic acid (EDTA) was described as a good titrant, either for calcium assay or for the measurement of magnesium [15]. Those are designed as complexometric titrations [18]. EDTA had good results when compared to previous techniques for being more accurate, good values for precision (maximum 0.058 mg of variation for inorganic samples) and recovery and for delivering faster results [19]. The disadvantage of titration techniques in biological samples is that it requires the previous removal of proteins [19].

For magnesium, potentiometric titration is usually performed, which turns out to be a titration that uses electrodes: an indicator electrode and a reference electrode (usually silver), with the potential difference measured through an EDTA titration [19].

### 4.3. Flame Emission Specrtophotometry

**Figure 1:**
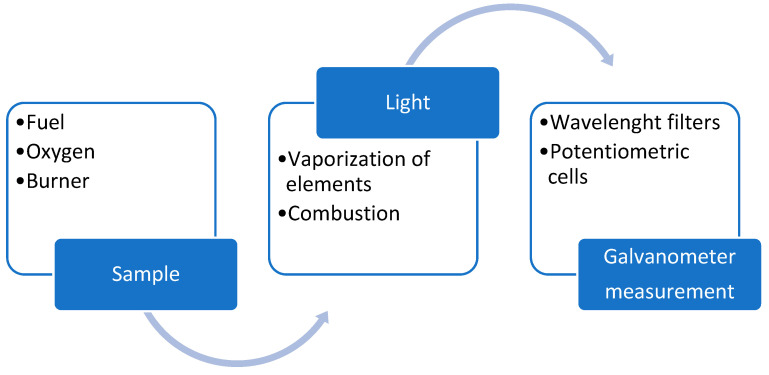
Diagram explanation for the methodology of flame emission spectrophotometry.

First used for this purpose in 1946, this method was one of the first used to determine concentrations of sodium, potassium, and even lithium in samples of blood and urine [20]. In this technique, the solution under analysis is placed into a chamber and is directed towards the flame [20]. The light produced by the vaporization of elements (which combusts) is conducted through filters to a potentiometric cell, which activates a galvanometer. Ion concentrations are then estimated from the galvanometer reading [20]. This procedure is succinctly outlined in Figure 1.

For urine samples, they must be diluted with water before determination, since there are different dilutions required for the assay, for each ion in particular [20]. When there are particles in the suspension, the sample must be filtered before processing [20]. The great advantage of this method is that it is faster to obtain the results, when compared with methods used previously. Unfortunately, for this method, it is necessary to use a large amount of sample, especially for potassium determination [20]. Another disadvantage is that the pressure must be very well controlled so that air bubbles do not enter the solution as the process progresses, which has been solved with the use of more modern equipment. The great disadvantage pointed out for this method is the need for a constant and uninterrupted gas source to keep the flame going [20]. In addition, this method presented undesirable values for accuracy, exceeding 3.5 percent of the amount present in the samples [21].

Flame photometry was also one of the first techniques to be used for the determination of magnesium. In 1958, some authors experimented with this methodology for the determination of calcium and magnesium in food, and an article was later published referring to this technique as the most suitable for the determination of these ions in plants [22]. This method eventually began to be used in biological samples, but the results were systematically unsatisfactory as there were too many interferents that needed to be eliminated in order to rely on this test [21].

This method was also used in several studies with the aim of measuring intracellular ions in the 1960s and later in the 1980s, however, not in human fluids. Estimates were made for intracellular potassium and sodium concentration in bacteria using flame photometry, with *E. coli* cultures, through a calculation that included the value of water inside each bacterium, density of the bacteria pellet, and estimated extracellular space [23].

### 4.4. Fluorimetry

In 1965, some fluorometric assays were performed for the determination of calcium in body fluids [24]. With this analysis, a fluorescent complex is formed at alkaline conditions upon the presence of calcium ions, when excited to a wavelength of 436 nm [25]. This methodology has the advantage of not requiring the elimination of protein interferents or magnesium ions that could complex with the fluorescent marker [25]. However, this method suffers a great amount of interference from temperature, bilirubin, and haemoglobin, and it presents many limitations for urine samples [25]. For this method, the precision and accuracy values for calcium assay in biological samples do not seem to meet the requirements recommended for medical application [25].

This technique was also applied for intracellular calcium and magnesium determination experiments, but the results were far from expected, with several interferences with other ions and biological artifacts [26].

### 4.5. Potentiometry

The increasing clinical necessity of assessing the concentration of ions in body fluids leads to the development of new techniques that are easier and faster in response. One of these methods is potentiometry. This assay is based on measuring the potential between two electrodes in two separate phases, in the absence of a current [27]. The electrodes can be made of various types of materials with electrical properties, allowing the measurement of ion concentration [28]. The reference electrode, whose potential is known, is the one from which the indicator electrode potential is measured (the response depends on the concentration of the analyte to be measured) [27].

More recently, several selective electrodes have shown great utility and a good performance regarding the determination of serum and urinary ionogram when compared to flame photometry, especially due to the possibility of determining several ions at once with multisensor systems [29,30]. For this case, there are electrodes with sensors for the different ions in a chamber where the sample is measured. Measurement results are compared to the reference electrode sensor (blank) [31]. Since the reference electrode potential is constant, measuring the potential developed by the sensor makes it possible to obtain the amount of analyte present in the sample, according to the Nernst equation [8].

For each sensor, a calibration must be performed for each type of fluid we intend to analyse. Electrodes should be as sensitive and specific as possible, and the signal obtained is generated by the charge difference between the two phases, which can be separated through a membrane [28].

**Figure 2:**
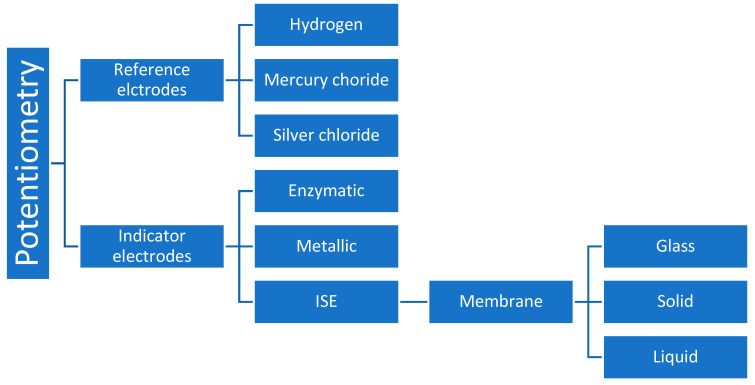
Diagram representation of the various types of the most used materials for potentiometric analysis.

A wide variety of sensors and membrane electrodes have been developed in order to solve some analytical problems. Reference electrodes can be hydrogen based (in disuse due to the various difficulties of use and interfering), mercury chloride (very sensitive to temperature variation), or silver chloride (most used because it has less interference) [32]. In addition to having a known potential, reference electrodes must be constant and not suffer interference by the solution under study [31]. Indicator electrodes can be enzymatic (used to quantify molecules such as glucose), metallic, or ion selective electrodes (ISE), which are most used in an ionogram [7,33]. As for the last ones, there can be glass membrane electrodes, solid membranes, or liquid membranes [28,34]. Figure 2 represents the variety of materials that are most used in potentiometry. The membrane, which separates the two phases, must have minimal solubility, allow electrical conductivity, and bind selectively to the analyte [28]. For glass membranes, it does not suffer interference from oxidants or reducers and can be used on a wide range of pH levels. It is very useful in the determination of cations. These kinds of membranes can be acquired in various formats (lamp shape, flat membrane, miniature adapted to needles, etc.) [35].

Solid membranes are composed of insoluble inorganic salts and can be used for anions and cations. They are widely used for ionograms and gases in blood and urine determinations [28]. In addition to being simple to use, there are membranes that offer great versatility, depending on the polymers used as constituents: there can be incorporated carbon materials, redox complexes, or other intercalating components to enhance the specificity of the membrane according to the target analytes [28].

For liquid membranes, there is a dissolved ionophore (or carrier) in an organic slimy membrane [28,34]. The ionophore allows the passage of the desired analyte through the membrane. This type of membrane is traditionally used for measuring calcium, and it is a very selective one [25]. Calcium electrodes are widely used in potentiometry: they are liquid membrane electrodes with aliphatic phosphoric diester acids dissolved in a polar solvent, allowing the exchange of cations. The internal reference solution, in contact with the membrane, contains a known concentration of CaCl_2_ and an Ag/AgCl electrode [36]. A potential develops across the membrane, which is the result of the difference in calcium ion activities between the two solutions. To obtain the calcium value of the sample, a calculation based on the Nernst equation is applied [36].

Recently, there have also been tested pseudo-liquid contact membranes for ion determinations with potentiometry using a formation of a polyethyleneimine/polystyrene sulfonate complex for sodium and potassium in human serum and urine, resulting in faster results and without previous preparation of the sample [37].

Initially, potentiometric tests in urine were carried out on undiluted samples, but discrepant results were obtained when compared to photometry of flame, especially for potassium measurement. Later, with optimization of the technique, it was stipulated that urine samples should be diluted [38]. This method’s advantage relies on its easiness to use, without needing a flame, and its versatility concerning the type of sample, with results in few minutes and at a low cost. The downside is just the fact that it needs a source of electricity to function [31]. Currently, potentiometric methods used in clinical practice for an ionogram have accuracy and precision values ranging from 0.1 to 3% for biological samples, and results can be obtained in few minutes in automated equipment [7]. New studies point to even faster results with the use of new sensors: in 2023, Nelson et al. obtained results for urinary chloride concentration in seconds, with a chronopotentiometric sensor [39]. A new approach for measuring ions was described recently using a differential microfluidic sensor with a frequency reading of microwaves and compared with results obtained by potentiometry. The results seemed to be promising and fast, using horse urine samples [40].

Use of potentiometry has also been reported for assays of various ions inside cells, using microelectrodes, but never on urinary epithelial cells. In 1984, experiments were described for the determination of an intracellular ionogram in cells of frog skin epithelium, but it was only possible to infer the intracellular activity of the ions, not their concentration [41]. Other experiments have been performed over the years to measure intracellular ions, using mostly glass membrane microelectrodes in plant cells. However, this type of measurement has as limitations the fact that ionic activity is reported only at one point in the cell and cannot be used in tissues because neighbour cells will have to be destroyed to reach the target layer [42].

### 4.6. Chromatography

Ion exchange chromatography enables a separation based on molecule charge differences as they interact with a matrix. This matrix can be column-shaped and is called stationary phase, while the liquid sample is called mobile phase [43].

Some authors have found this technique useful for the determination of ions in organic fluids such as urine because it can measure several parameters at once and because it requires small volumes of a sample. However, it is not generally used in clinical practice as it is an expensive and quite complex system, especially due to the need for several dilutions of samples, leading to a much longer response time than using potentiometry [44,45].

A new approach combining chromatography with a conductimetric detector using mass spectroscopy was recently published for the determination of ions in urine, showing good values of accuracy and precision and with no significant matrix effects [46].

### 4.7. Electrophoresis

Electrophoresis is a technique where ions from a sample move under the influence of an applied voltage. This mobility process depends on the molecule to be analysed as well as its viscosity, size, and also the voltage that is applied to the medium where it is going to move [47]. So, neutrally charged molecules will not be able to move as only the ions will be affected by the electric field. Electrophoresis has several variants according to the materials and media that can be used in the system, but the most used is capillary electrophoresis as it provides results with good separation in a relatively short time [47].

In capillary electrophoresis, there is a high-voltage electrical system, a place for the introduction of the sample, a capillary tube, a detector, and a reading system [47]. A disadvantage for this method is that the application of an electrical charge generates an increase in temperature, so some systems must be equipped with temperature control devices to enable the reproducibility of results [48]. Inorganic ions are easily separated using this methodology, given their low molecular weight. Chlorine ion has been determined by this technique by some authors over the years, and some limitations for those experiments were pointed out, such as difficulties in the separation at low pH or in high-saline solutions [49,50]. There are some studies that compare the use of capillary electrophoresis with ion exchange chromatography for the determination of ions in biological samples [51]. Some authors consider that chromatography allows greater versatility of samples and offers greater confidence in results when compared to electrophoresis [45]. A 2000 study also considered that both techniques could be complementary, suggesting the application of an ion exchange polymer in the electrolytic capillary, allowing the separation of compounds according to their ionic interactions with the polymer and with its electrophoretic mobility [51]. Capillary electrophoresis can also be used for the determination of calcium and magnesium [52].

In order to increase the performance of ion analysis in high electric conductivity solutions, Lancioni et al. recently developed a new background electrolyte for capillary electrophoresis, with detection through indirect UV absorption [53]. The results were very promising, but the procedure only tested for inorganic samples [53].

Although some authors refer to its usefulness in samples from organic fluids and tissues, this technique, regarding the determination of calcium and magnesium, is currently most used for the analysis of water and food [49,52].

### 4.8. Kinetic Colorimetric Tests

Colorimetric tests are the most used for the determination of calcium and magnesium in blood and urine. It is one of the oldest methods (1950s and 1960s) for these parameters, but it has been optimized throughout the years and is widely used in hospital laboratories, in automated analysers [54]. For this kind of assay, there is a chemical reaction that leads to a colour change, when one substance meets another one with a known colour (“blank” of the reaction) [55]. According to the intensity of the colour, the concentration of the substance can be determined. Initially, the results were observed by the naked eye, but, nowadays, a spectrophotometer is used for more sensitive and consistent readings [55]. According to the reaction chemistry, the result can be displayed in a graph, which provides the colour intensity information based on the wavelength of the light source [55]. Therefore, these tests are based on the measurement of absorption variations by spectrophotometry in the visible zone (between 400 and 700 nm) [54].

For calcium determination, the cresolphthalein method, described by Kessler and Wolfman, was widely used: the reaction product had a maximum absorption at 575 nm (violet colour complex) but it demonstrated many interferences with the presence of magnesium [56]. In the methylthymol-blue method, whose chemical structure is very similar to cresolphthalein, a calcium complex with maximum absorption is obtained at 612 nm [57]. However, there is the inconvenient factor that those reagents are not very stable and sensitive to small variations in temperature [25]. The Arsenazo III technique has been the most used in the last years for the measurement of calcium in blood and urine: at the end of the reaction, a blue complex, whose intensity is proportional to the calcium concentration in the sample, is formed, and the absorbance of the reaction product is measured between 600 and 680 nm [58]. Recently, an automated colorimetric assay was also developed using the chromophore compound 5-nitro-5′-methyl-(1,2-bis(o-aminophenoxy)ethan-N,N,N’,N’-tetraacetic acid (NM-BAPTA) and, in a second reaction step EDTA, with the calcium concentration being directly proportional to the absorbance measured photometrically [59]. This method presents very high precision values with variations between 1.1 and 3.1% for calcium in human urine, and results can be obtained in a few minutes in automated equipment [9].

For the determination of magnesium concentration in biological samples, Lindstrom and Diehl first suggested the use of o,o’-dihydroxyazo as an indicator [60]. However, with this reagent it was concluded that, for urine samples, results are quite unreliable and it is necessary to carry out two measurements at different wavelengths due to interferents [60]. Many other indicator reagents were used in these determinations, such as “eriochrome black T”, “fast blue BG”, “8-hydroxyquinolone”, or titan yellow, and most have fallen out of use because of their various limitations [10]. Note the importance of the latter for the determination of magnesium, which has been considered one of the best options for non-automated analysis, demonstrating very low variation coefficients [10]. Methylthymol blue was also a widely used indicator for the measurement of magnesium with lower variation coefficients than titan yellow. However, it suffers some interferences with the presence of bilirubin in the sample and it has limited linearity [61]. Nowadays, the most used colorimetric test for magnesium measurement in blood and urine is the xylidyl method [10]: magnesium ions form pink complexes with xylidyl blue in an alkaline medium, with a purple intensity (measured at 505 nm) directly proportional to the magnesium concentration in the sample.

In the xylidyl method it is also possible to add a complex capable of eliminating the interference of calcium ions in the reaction [10]. This method presents very high precision values with variations between 0.8 and 2.1% for magnesium in human urine, and results can be obtained in few minutes in automated equipment [12]. Regarding the use of these methods for the intracellular analysis of calcium and magnesium, it dates to 1928 when some authors injected a dye (alizarin) in parasite cells for the microscopic observation of intracellular calcium granulations [62]. In the year 1966, other investigators performed the same procedure with the murexide dye for muscle cells [63]. Arsenazo III was also used in this regard but, upon microscopic observation, it was found that its distribution in the cell was not uniform. Although innovative for the time, this method did not allow for quantitative analysis [58].

### 4.9. Enzymatic Colorimetric Assays

Very similar to kinetic colorimetric assays, with enzymatic assays the analyte is a substrate that will be catalysed by a specific enzyme [64]. The product of a first enzymatic reaction can also be taken up by another enzyme that is coupled to a colorimetric precipitate [64].

This method has been used by some authors for sodium and potassium determinations in serum, but their results were statistically different from the values obtained by potentiometry [64]. For sodium, β-D-galactosidase and O-nitrophenyl-β-galactopyranose as the substrate were used; for potassium, they used phosphoenolpyruvate, pyruvate kinase, and lactate dehydrogenase [64].

For the determination of calcium in serum, the method was first described by Kimura et al., performing a reaction in which calcium inhibits the activity of the enzyme and the reaction compound produced was inversely proportional to the concentration of calcium in the sample [65]. For magnesium, this type of test has been widely used and automated, especially using glucose-6-phosphate (G6P), nicotinamide adenine dinucleotide phosphate (NADP+), glycerol kinase, glycerophosphate oxidase, and peroxidase [10]. However, these methodologies are not commonly used in routine laboratories as response times are longer than with kinetic colorimetrics [10]. The main problem that this type of testing can present in terms of accuracy is the variation in the matrix used. Therefore, special attention must be paid to the homogeneity of the colour [55].

### 4.10. Flow Cytometry

Flow cytometry is a useful technique to check for components or markers inside or on the surface of various types of cells [66]. It has already been used for the determination of intracellular potassium and sodium but never in urinary cells. With this method, a multi-parametric result can be obtained for each cell element in a solution. Through laser lights, flow cytometer equipment is capable of graphically showing the signals obtained either by light scattering, which will vary according to the size and complexity of each cell, or by fluorescence emission, which marks the components to be studied [66]. Fluorescence reagents used with cytometry are usually antibodies conjugated to a fluorescence marker, DNA markers, viability markers, protein-binding markers, and markers that bind to ions [67].

In 1997, a cytometric method for the determination of intracellular potassium was described in human and animal tissues and in cell lines, with satisfactory results demonstrating high sensitivity [68]. For the determination of intracellular calcium, this methodology has also been used: in 1994, some authors published their determination of calcium in rat myeloma cells using flow cytometry [69]. They described some difficulties such as working at very low concentrations [69]. However, since then, there have been on the market several new probes for this purpose and the optimization of this technique has evolved favourably [67,70].

This methodology allows performing measurements in cells individually, without requiring a large amount of sample, so it appears to be very promising for future studies.

### 4.11. Atomic Absorption

Atomic absorption spectrometry (AAS) is a spectroanalytic technique for the determination of elements through the absorption of radiation by free atoms in their gaseous form and in their ground state [71]. For the analysis, there is a cloud of atoms on which electromagnetic radiation strikes at a given wavelength, and the signal of its absorption is measured comparing it to the background absorption signal [71]. There are two types of atomic absorption techniques, according to the type of atomization used: flame and electrothermal. Flame atomic absorption is used for the determination of ions such as calcium, magnesium, sodium, and potassium [71].

A wide variety of samples can be analysed with this technique, such as tissues or organic liquids, plants, soils, waters, minerals, and technological materials [71].

For potassium ions’ determination, this methodology was used for the first time in 1987 in bacterial cytoplasm [14]. Later in 1991, Wood and LeMoigne performed new experiments with this technique for intracellular determination of sodium and potassium in fish cells [72]. Although it may be possible to apply it in human urine cells, this technique has the disadvantage that it destroys the cells and does not allow the ions to be assayed in each cell individually [71].

Atomic absorption spectrometry can also be used for the determination of calcium and magnesium. For calcium, the optimization of this technique had its beginnings in the 1960s and, over the years, some ways were developed to minimize the problems of calcium complexation with proteins, phosphates, or other compounds [73,74]. For magnesium, the use of this technique was first proposed in 1955 and has been successfully optimized over the years [10]. For both parameters, high specificities and sensitivities have been accomplished [73]. Since both ions are frequently analysed in the same samples, there are electromagnetic radiation sources already prepared for their application together [73]. Although it is recommended by many authors as a reference technique due to its accuracy, this methodology is not commonly used in hospital laboratories as it presents great costs for implementation and maintenance, as well as requiring very experienced professionals. In addition to this, the potential biological risk for those working with atomic absorption is high since it implies proximity to flammable and explosive gases [71].

### 4.12. Plasma Emission Spectrometry

This is a multi-element type of analysis that can also be used for urine samples [75]. With this method, the use of high temperatures leads to the formation of extremely ionized gases including ions in exited electronic states, which is called plasma. When those ions relax, light is emitted and detected [76]. However, this technique presents a complex procedure due to some limitations regarding the presence of easily ionised elements in the matrix of urine samples [75].

### 4.13. Paper-Based Devices

A new method for determining urinary ions was recently described based on the principles of paper chromatography, but using fluorescent probes that bind to ions, with a fluorescence reading device through a smartphone [77]. These authors performed their tests on fluids that simulated human urine, and the results indicated that the technique can be properly optimized for real urine samples, presenting just a few limitations regarding sodium detection limits [77]. This method presents the advantage of having fast and frequent screenings for the concentration of the ions throughout the day, on occasional urination, and it is very user friendly [77]. Although they show very promising results, these investigations are still very recent and will need further studies using real urine samples.

## 5. Discussion

**Figure 3:**
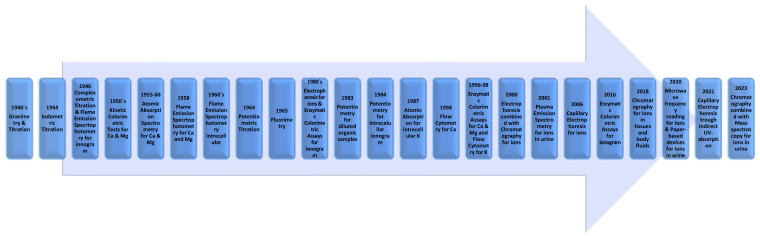
Chronological scheme that reflects the appearance of the different quantitative methodologies addressed over time.

With the results obtained in this review, it is possible to assume that there was a growing concern with precision and specificity values over time, especially when the use of titration methods emerged and interferents on other assays were attenuated with new studies and new protocols. Methods showing undesirable accuracy results concerning clinical analysis, like flame emission spectrophotometry or fluorometry, are not currently used in hospital laboratories, although they can be used in an investigation context. In addition, there was a growing interest in obtaining faster results, with the advancement of medicine and hospital diagnostics. It was possible to observe that an ion analysis went from many hours to a few minutes in about two decades, which may have reflected the needs of new organizations in health systems. Methods that are very time consuming, like gravimetry or chromatography with or without electrophoresis or enzymatic colorimetric assays, or present many interferants with biological samples, like titration without the complement of potentiometry and plasma emission spectrometry, are also not much used in clinical contexts, as they would need to be improved. In Figure 3, there is a chronological scheme that reflects the appearance of the different quantitative methodologies addressed over time.

In recent years, the emergence of more sophisticated technologies has allowed more and more reliability in the results but, in some cases, resulting in complex reactions that imply some risk for professionals, as with the case of atomic absorption techniques. For this reason, there is tremendous concern for the safety of health professionals, even seeking the use of simpler techniques, always evaluating the risk–benefit of their performance, as well as the associated costs. Flow cytometry (for intracellular purposes) and paper-based methodologies seem to be promising, but further studies and advances in those methods are being performed for clinical contexts. Potentiometric and colorimetric kinetic assays are nowadays widely used in automated equipment in healthcare systems for the determination of ionogram, calcium, and magnesium in biological samples.

## 6. Conclusions

Given the importance of ion determinations in human urine for diagnosis and therapy in the most diverse pathologies, it is interesting to verify the development of laboratory test methods for this purpose over the years. From the simplest and most archaic methods to the more time-consuming and costly, there is currently a great variety of options at the level of scientific investigation for the measurement of urinary ions, depending on the purpose of the study. However, at clinical and hospital levels, the trend will always be to improve the test performances and speed in obtaining results without excessively increasing costs, the complexity of the test, or the risks for the operator. In this sense, much has been advanced regarding free ions in 24 h urine, but there is still some way to go concerning the importance of intracellular ions in this type of sample as well as the use of occasional urine for monitoring these parameters.

## Abbreviations

ATP	Adenosine triphosphate
EDTA	Ethylene-diamino-tetraacetic acid
G6P	Glucose-6-phosphate
NADP+	Nicotinamide adenosine dinucleotide phosphate
AAS	Atomic absorption spectrometry
